# Reading the DNA of telomeres

**DOI:** 10.7554/eLife.107648

**Published:** 2025-06-18

**Authors:** Shamayita Roy, Claus M Azzalin

**Affiliations:** 1 Gulbenkian Institute for Molecular Medicine Lisbon Portugal

**Keywords:** telomeres, ssDNA, dsDNA, END-seq, alternative lengthening of telomeres, cancer, Human, Mouse

## Abstract

Experiments with tools designed to detect DNA damage reveal unique and conserved features of telomeres in cancer cells.

**Related research article** Azeroglu B, Wu W, Pavani R, Sandhu R, Matsumoto T, Nussenzweig A, Lazzerini Denchi E. 2025. Conserved and unique features of terminal telomeric sequences in ALT-positive cancer cells. *eLife* **14**:RP106657. doi: 10.7554/eLife.106657.

To proliferate uncontrollably, cancer cells need to maintain the length of their telomeres, regions of DNA that act as protective “caps” at the ends of chromosomes. In most healthy somatic cells, telomeres shorten slightly after every cell division. Once a finite number of divisions has been reached, telomeres become critically short and halt any further proliferation, creating a barrier to cancer development (Harley et al., 1990; Jones-Weinert et al., 2025).

Approximately 85% of human cancers overcome this barrier by activating telomerase, an enzyme that extends telomeres but is not normally expressed in somatic cells. The remaining cancers rely on a specialized DNA repair pathway called Alternative Lengthening of Telomeres (or ALT for short), which elongates short telomeres by copying DNA from a longer telomere within the same cell (Dilley et al., 2016; O’Sullivan and Greenberg, 2025).

Telomeres consist of two strands of DNA, which each contain a specific sequence of base pairs: TTAGGG and its complementary strand CCCTAA. At the end of the telomere, the strand rich in the base guanine (G) extends past its complementary strand to form a single-stranded region of 50–200 bases, known as the G-overhang (Figure 1; Jones-Weinert et al., 2025). While the general structure of telomeres is well characterized, much less is understood about how the telomeres of ALT-positive cancer cells differ from those that rely on telomerase (ALT-negative cells). Now, in eLife, Eros Lazzerini Denchi and colleagues at the National Cancer Institute – including Benura Azeroglu as first author – report repurposing an established DNA sequencing method to investigate key features of telomeres in these different types of cancer cell (Azeroglu et al., 2025).

**Figure 1.**
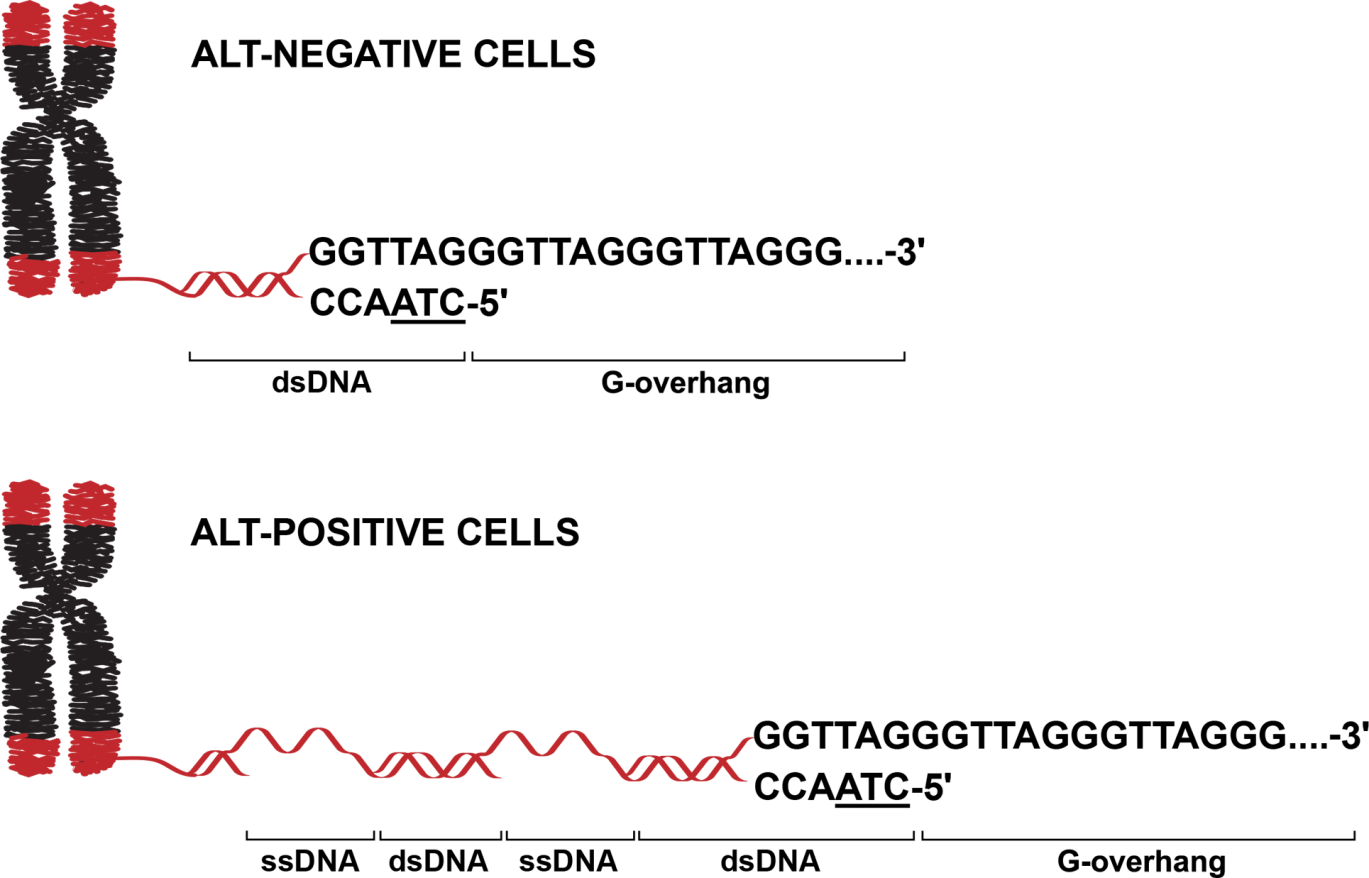
Unique and conserved telomere features in cancer cells that use different telomere maintenance mechanisms. In cancer cells, the telomeres (red) covering the end of chromosomes (black) are maintained and lengthened by either the ALT pathway (ALT-positive cells; bottom) or the enzyme telomerase (ALT-negative cells; top). ALT-positive cells, ALT-negative cells and non-cancer cells (not shown) share several features: a conserved 5’ terminus ending with the base pair sequence ATC (underlined), and a G-overhang at the 3’ terminus. The telomeres in ALT-positive cells also contain regions of ssDNA that are not found in ALT-negative cells or non-cancer cells. The method employed by Azeroglu et al. did not establish whether the ssDNA within ALT telomeres is C- or G-rich. ALT: alternative lengthening of telomeres; ssDNA: single-stranded DNA; dsDNA: double-stranded DNA.

The team employed END-seq, a powerful sequencing method which was originally developed to map double-stranded breaks across the genome (Wong et al., 2021). Since the end of chromosomes resemble one end of a double-stranded break, END-seq should identify telomeres as unidirectional reads rather than the bidirectional reads that characterize typical double-stranded breaks. By using this technique on human cell lines that express telomerase, Azeroglu et al. confirmed it could identify ‘telomeric reads’, which were defined based on whether they were unidirectional and contained at least four consecutive TTAGGG (G-rich) or CCCTAA (C-rich) repeats. Almost all of the identified telomeric reads derived from the C-rich strand, as would be expected when looking at telomeres as one-ended double-stranded breaks, confirming the robustness of the approach.

Applying this technique to a variety of cancerous and non-cancerous cell lines as well as mouse tissues revealed that the ends of chromosomes in ALT-positive cells are remarkably similar to those in ALT-negative and non-cancerous cells. In all cases, the 5′ end of the telomeres typically terminated with an ATC sequence (Figure 1). Loss of the telomere-binding protein POT1 – which regulates the 5’ end sequence at the end of chromosomes in ALT-negative cells (Hockemeyer et al., 2005) – also altered the telomere sequence of cancer cells that rely on the ALT pathway. This is a striking finding given that telomeres elongated by ALT are typically considered to be structurally distinct: they are highly heterogeneous in length, possess long G-overhangs, and are enriched in unusual secondary structures (Arora et al., 2014; O’Sullivan and Greenberg, 2025; Yang et al., 2021). Yet, despite these structural differences, the findings of Azeroglu et al. suggest that key features in the organization of telomeres are conserved across ALT-positive and ALT-negative cells.

This raised the question of whether ALT-positive telomere DNA sequences have other unique features that distinguish them from those lengthened by telomerase. To explore this, Azeroglu et al. took advantage of S1-END-seq, a newer version of END-seq that can detect internal stretches of single-stranded DNA (ssDNA) within telomeres (Matos-Rodrigues et al., 2022). This approach identified frequent internal ssDNA regions within the telomeres of ALT-positive cells. Although this method did not reveal whether the ssDNA regions are C-rich or G-rich, it clearly demonstrated that they are a hallmark of ALT-positive cells, as these regions were virtually absent from telomerase-positive cancer cells and non-cancer cells (Figure 1).

The study by Azeroglu et al. has several important implications. First, it supports the notion that the telomeres of ALT-positive cells are inherently damaged or incompletely replicated, as the presence of internal ssDNA regions likely reflects defective telomere replication (Mazzucco et al., 2020). Second, the S1-END-seq technique offers a robust way to distinguish ALT-positive from ALT-negative cancers. Translating this method to clinical settings could improve how well tumors are classified and aid in stratifying patients for personalized treatments. Third, studying how ssDNA regions are formed and maintained in ALT-positive cells may uncover vulnerabilities that could be exploited therapeutically. For instance, ssDNA is inherently unstable and, if unprotected, can lead to DNA breaks, genome instability and cell death. Future studies should focus on scaling up S1-END-seq to identify the factors that are shielding these fragile ssDNA regions and develop drugs that specifically disrupt their function.

Taken together, the work of Azeroglu et al. highlights that, sometimes, less is more. While the scientific community continues to push boundaries to develop new tools to dissect the biology of cancer cells, these findings demonstrate that repurposing existing methodologies can yield important insights.
